# A Survey of Lost-in-Space Star Identification Algorithms Since 2009

**DOI:** 10.3390/s20092579

**Published:** 2020-05-01

**Authors:** David Rijlaarsdam, Hamza Yous, Jonathan Byrne, Davide Oddenino, Gianluca Furano, David Moloney

**Affiliations:** 1 Intel Corporation, Intel R&D Ireland Ltd., Collinstown, Collinstown Industrial Park, Co. Kildare, W23CW68 Collinstown, Ireland; hamza.yous@intel.com (H.Y.); jonathan.byrne@intel.com (J.B.); david.moloney@intel.com (D.M.); 2 European Space Agency/ESTEC, 1 Keplerlaan 2201AZ, 3067 Noordwijk, The Netherlands; Davide.Oddenino@esa.int (D.O.); gianluca.furano@esa.int (G.F.)

**Keywords:** star identification, attitude estimation, star tracker algorithms, star feature extraction

## Abstract

The lost-in-space star identification algorithm is able to identify stars without a priori attitude information and is arguably the most critical component of a star sensor system. In this paper, the 2009 survey by Spratling and Mortari is extended and recent lost-in-space star identification algorithms are surveyed. The covered literature is a qualitative representation of the current research in the field. A taxonomy of these algorithms based on their feature extraction method is defined. Furthermore, we show that in current literature the comparison of these algorithms can produce inconsistent conclusions. In order to mitigate these inconsistencies, this paper lists the considerations related to the relative performance evaluation of these algorithms using simulation.

## 1. Introduction

Accurate attitude determination for spacecraft remains a relevant and often researched topic due to the progress in spacecraft technology of the past few decades. Significant developments have taken place since the 2009 survey by Spratling and Mortari [1]. Advancements in technical components such as microprocessors, optical systems, and algorithms enable more extensive capabilities in attitude determination. Meanwhile, the requirements for attitude determination are becoming even more strict: communication, high-resolution observations and other applications require more accuracy than ever before.

Even though there are numerous sensor options to determine the attitude of a spacecraft, the most accurate sensor is the star sensor [2,3], which is becoming the most widely applied sensor in spacecraft attitude determination [4]. The classical star sensor is a relatively complex system and was until recently used only in high-end missions. However, with the rise of CubeSats and other low-cost satellites, some low-cost options have emerged [5,6]. Arguably, one of the most important components of the star sensor system is the star identification algorithm. This algorithm is part of the data processing function of the star sensor and identifies which stars are present in a specific scene.

In their extensive survey, Spratling and Mortari identify two top-level categories of star identification algorithms: lost-in-space algorithms and recursive algorithms [1]. The first category is able to autonomously identify the stars present in a scene without prior attitude information, solving the so called lost-in-space problem. The second category is a more efficient star identification algorithm that can be used when some attitude information is available. This paper covers the first category, since solving the lost-in-space problem is the most critical function of the star sensor system. Numerous categorizations have been made for the feature extraction phase of the identification algorithm. Padgett [7] defines two categories for the feature extraction phase, namely: subgraph isomorphism based feature extraction and pattern association feature extraction. Algorithms of the first category approach the star identification problem by considering the stars as vertices in a subgraph, with the angular distances between the stars as edge weights. To identify the stars, an isomorphic subgraph has to be found in a database. These algorithms include the polygon angular matching algorithm [8], triangle algorithms [9], the color matching algorithm [10], group match algorithms [11], and the pyramid algorithm [12]. Algorithms of the second category assign each star a pattern based on the relative positioning to neighbouring stars and try to find the closest match to the measured pattern in the pattern database. These include grid algorithms [13], the singular value method algorithm [14], the Log-Polar transform algorithm [15], the Hidden Markov Model based algorithm [16], the genetic algorithm based identification algorithm [17], the K-L transformation algorithm [18], the ordered set of points algorithm [19] and the labelling technique algorithm [20]. Although some papers suggest a third category for novel algorithms using techniques such as neural networks or genetic algorithms, these can also be classified using the categories above since this classification is based on feature extraction methods [21].

Since star identification algorithms have different working principles and their performance is dependent on application environment, direct comparison of performance metrics between the algorithms is challenging. Inconsistencies exist in current literature with respect to performance metrics. Therefore, a structural approach to measuring relative algorithmic performance is required.

In this work, we shall first describe the star sensor system and the functional flow of the star sensor. Then, recent lost-in-space star identification algorithms are surveyed and their characteristics are compared in terms of application environment and analytical performance. Lastly, considerations for a structural approach to comparing star identification algorithms are presented.

### Methodology

In this survey, lost-in-space algorithms are surveyed that have been published after the publication of the 2009 survey by Spratling and Mortari. Algorithms that were published before 2009 but not covered in the previous survey that have proven to be influential in the field of lost-in-space star identification algorithms have also been included. In total, 104 relevant publications were identified, out of which a selection was made based on number of citations, quality of the paper, and representativity of the field. The aim of this paper is to give a qualitative representation of the current state of the art in the field. To this end, a selection of 14 lost-in-space algorithms was made.

## 2. The Star Sensor System

**Figure 1:**
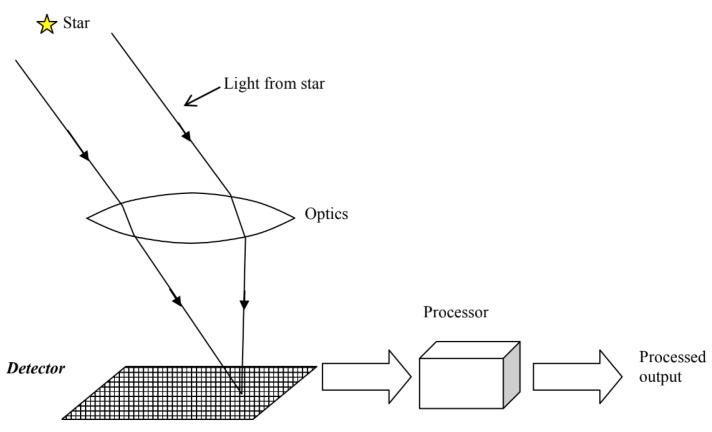
Schematic generalised star sensor model (reprinted from ECSS-E-ST-60-20C [22] with permission of the European Space Agency, the owner of the copyright on behalf of ECSS).

According to the ECSS-E-ST-60-20C standard [22], a star sensor has three functions: imaging, detecting and data processing (see Figure 1). ECSS-E-ST-60-20C identifies three different types of star sensors, e.g., the star camera, star tracker and the autonomous star tracker. Each type of star sensor is classified by the minimum and additional capabilities of the system and has a defined set of inputs and outputs. This paper is concerned with lost-in-space algorithms, and thus with the autonomous star tracker.

**Figure 2:**
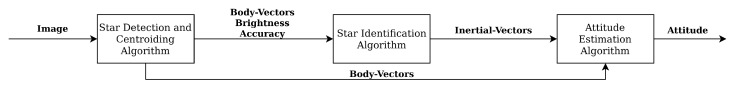
Functional flow star sensor.

The autonomous star tracker is capable of autonomous attitude determination and autonomous attitude tracking. It captures an incoming signal (i.e., star light), processes this signal and provides a number of minimum outputs defined in the standard. The star identification algorithm is an integral part of the star sensor functional flow, as shown in Figure 2.

The input of the star identification algorithm is typically the body-vectors, accuracy and the brightness or magnitudes of the stars present in the image. These are found using a star detection and centroiding algorithm which detects the stars present and determines their position in the image frame with sub-pixel accuracy. While the absolute magnitude measurement is a less accurate parameter due to the noisy detector sensitivity, the relative magnitudes of stars in one image can be used more accurately in the identification process. The star identification algorithm uses the position and in some cases magnitude measurements to extract features which are used to search the on-board database for possible matches. After finding the probability of error of the solution, the output of the star identification algorithm is the inertial vectors of (some of) the stars in the image if the identification is successful. Otherwise, a flag indicating that the identification has failed is raised. It is important to note that failure of star identification is considered less of an issue than false positive matching, which could be a threat to the spacecraft. Once the lost-in-space problem has been solved, the system can switch to a faster recursive identification method by using the previously found attitude estimation for identifying the body vectors.

After the star identification algorithm, an estimator such as Davenport’s q-method, QUaternion ESTimator (QUEST), FOAM, EStimators of the Optimal Quaternion (ESOQ and ESOQ2), or the Singular Value Decomposition are used to calculate the translation between the body vectors and the inertial vectors [23].

### 2.1. Input Noise on the Body Vector

**Figure 3:**
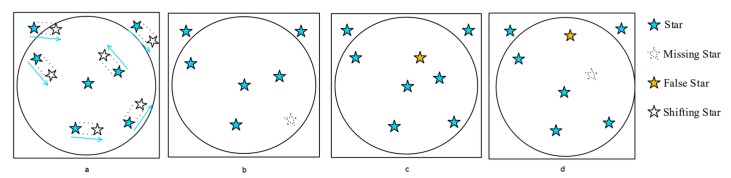
Star identification noise factors. (**a**) star shift; (**b**) dropped star; (**c**) false star; (**d**) combination of noise factors (adapted from [24]).

The star sensor system is subject to multiple types of noise in measuring the body vectors, as shown in Figure 3.

Firstly, external factors such as reflective objects, satellites, dust, etc. might cause the sensor to measure false stars. Furthermore, radiation effects such as a Single Event Upset (SEU) might cause the detector to be saturated at certain points, also causing false star measurements. Stars that are not in the on-board database but are present in measurements are in effect also false stars: the algorithm is unable to identify these stars. Thermal deformations and other optical imperfections of the optical system can cause star shift, causing the star locations measured to translate from the ground truth. Partially blocked field of view (FOV), dead-pixels, or detector effects cause dropped stars. A combination of noise types can also be present. The star identification algorithm must deal with these types of noise and should be able to return valid attitude information when subject to the mission environment in terms of noise. Note that other noise factors not directly influencing the body vector (such as star magnitude, color, etc.) can affect the algorithmic performance as well in some cases.

### 2.2. On-Board Database

An integral part of the star identification algorithm is the on-board database that is used to store the star features for identification. This database is generated in from an existing catalog (such as the Hipparcos catalog [25] or the Bright Star Catalog (BSC) [26]) that includes star positions on the celestial sphere and magnitudes. The database contains the parameters used for identification of the stars and is structured such that entries can be accessed efficiently. A few pre-processing steps have to be taken into account when generating the database. Since the detector can only identify stars up to a certain magnitude, the catalog is filtered using a magnitude threshold. Stars that have centroids that are too close to each other (also called double stars) need to be excluded from the database because they can cause localisation and brightness determination to fail [7]. Since the celestial sphere does not have a uniform distribution of bright stars, the number of stars per sky partition can differ significantly. Therefore, in order to ensure proper attitude determination, it is necessary to verify that the database covers the minimum amount of stars needed for a successful identification within the FOV for all orientations. Database size is highly dependent on the FOV of the sensor system, as a smaller FOV implies a larger number of possible star patterns. The database is built up taking into account the chosen searching method and pattern parameters. Efficient searching algorithms for the star database include the *k*-vector technique by Spratling and Mortari [27] and hashing techniques [28].

## 3. Advances in Star Identification Algorithms

Multiple novel solutions have been proposed since 2009 and hardware has advanced significantly. In this section, we shall survey the relevant publications on star identification algorithms that are not covered by the Spratling and Mortari survey paper. The publications covered are published in a peer reviewed journal and improve performance significantly or have a novel, promising approach.

### 3.1. The Singular Value Method (SVM)

In 2003, Juang, Kim, and Junkins published their star pattern recognition algorithm method using singular value decomposition (SVD) [14]. This novel approach does not need a separate attitude determination algorithm, but rather produces the attitude directly, eliminating the last block in the functional flow of Figure 2. The algorithm uses a pattern association feature extraction. The pattern recognition is achieved recursively: the brightest star is selected as the boresight direction and four bright stars in the field of view are chosen. These vectors are decomposed and the singular values are compared with the database singular values. If the identification fails, another star is chosen as the boresight direction. If a match is found, the attitude is directly calculated from the singular values. In the original paper, testing is performed on only 10 real star images, achieving a matching rate of 70%. Even though Juang and Wang published a paper improving the algorithm in 2012 [29] by using a sensitivity analysis method and eliminating the brightness ranking, the testing of the algorithm is not extended beyond the 10 images. Furthermore, for some images, the algorithm needs many iterations to find a match.

In 2019, Wei et al. published a paper using the oriented SVD transformation of triangles for the star identification algorithm [30]. This algorithm also suffers from redundant matches and therefore requires a reliability evaluation using star voting. Wei et al. compare their algorithm against the grid algorithm by Padgett and Kreutz-Delgado [13] and the SVM algorithm and report that their algorithm outperforms both in terms of robustness against magnitude and positional noise with respect to the SVM algorithm, with the grid algorithm performing significantly worse. The robustness against false stars is similar with respect to the grid algorithm at around 93.2% for three false stars versus 93.8%, while the SVM algorithm reaches only 70% with three false stars present in the image. The SVM algorithm outperforms both algorithms in terms of speed, while also outputting the attitude (skipping the need for an attitude determination algorithm and in effect reaching even better performance). This does come at a highly increased memory cost with respect to the other two algorithms. While the SVM paper does not specify a search method, it is reasonable to assume that the search time is O(n). The oriented singular value feature method uses a binary search for the initial matching, achieving O(logn) time while the original grid algorithm only achieves linear time O(n).

### 3.2. Modified Grid Algorithms

In 1997, Padgett and Kreutz-Delgado published a well-known paper on their grid algorithm, a lost-in-space type algorithm using a pattern recognition feature extraction [13]. This algorithm has relatively poor performance in terms of magnitude and positional noise and recently attempts have been made to mitigate these shortcomings. In 2008, Na, Zheng, and Jia published a paper on their elastic grey grid algorithm which significantly improves the performance of the original algorithm [31]. The grey grid algorithm eliminates the hard template matching of the original grid algorithm that caused it to be weakly robust to magnitude and positional noise. The hard template matching is replaced by a cost function that takes into account the difference between the measured pattern and the database pattern, and uses the relative magnitudes of the stars as a weight. This makes the pattern grey instead of binary, increasing the robustness to magnitude noise. The computational complexity increases with respect to the original algorithm to O(bn), where b is the number of stars in a pattern. The authors simulate the algorithm against the original grid algorithm, and achieve a better recognition rate than the original grid algorithm under both magnitude and positional noise and false star presence. The algorithm is, however, slower than the grid algorithm by about 26.6% and does not address the issue the original grid algorithm has with respect to the low probability of selecting the right reference star and rotating the reference grid by selecting a neighbouring (pivot) star outside a certain radius. According to [2], this probability can be as low as 50%.

In 2016, Aghaei and Moghaddam published a paper on an improved grid algorithm using a number of optimizations [32]. These optimisations include forming radiometric clusters based on the relative brightness of the stars in an image. This reduces the probability of choosing a false pivot star. Another optimisation that is introduced is similar to the gray grid algorithm. While a larger cell size obviously makes the algorithm more robust to positional noise, it also reduces the pattern resolution. In order to solve this trade-off, the grid cell sizes are optimised as a function of the standard deviation of the positional noise. Lastly, the match classification error is reduced using ‘vetoing’ by only keeping the two matches with the highest vote scores and discarding all other matches for a certain orientation. This reduces the probability of false positive or negative matching. In combination with this, the optimum threshold for rejecting voting scores corresponding to false matches is calculated using Bayesian decision theory. The algorithm achieves O(αn) time, where α is the number of pivot stars. The authors compare the improved grid algorithm against the original grid algorithm, and conclude that it strongly improves the robustness against positional noise and the presence of uncatalogued stars. The authors do not address the robustness against false stars.

### 3.3. Star Identification Based on Log-Polar Transform (LPT)

**Figure 4:**
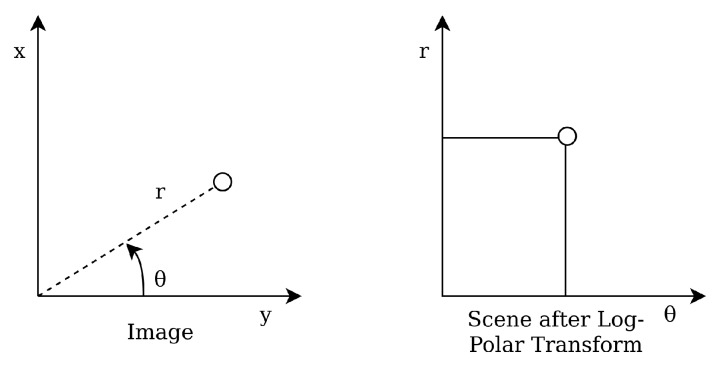
Log-polar transform.

Wei et al. presented their star identification algorithm based on the Log-Polar Transform (LPT) in 2009 [15]. It is another lost in space algorithm with a pattern based feature extraction. The LPT transforms the star patterns from Cartesian coordinates to polar coordinates with logarithmic radius (see Figure 4). The LPT properties are invariant to rotation and scale, and are calculated by shifting the star image such that guide star *t* is located at the origin. Then, the coordinates are transformed by a LPT and digitised as a *m* × *n* sparse matrix, with *m* being the sampling points in θ direction and *n* the sampling points in *r* direction. This result is projected on the θ axis in order to make a one-dimensional 1 × *m* vector *lpt(t)*. The same procedure is performed on the catalog stars. The algorithm then encodes the patterns as strings while reducing the sparsity of the vector and matches these to the database stars.

By encoding the patterns as a string, searching for the right pattern in the database is analogous to searching for the most similar word in a dictionary. The LPT algorithm uses a modified string matching algorithm based on Knuth–Morris–Pratt (KMP), which keeps track of how many characters in a string match with the particular database section and how many are mismatched, while maintaining a search range limitation. KMP has complexity O(n) . If certain threshold conditions are met with respect to these values, a match is found. Otherwise, the algorithm is iterated on another star *t* in the image. If no star produces a match, the identification fails. Wei, Zhang, and Jiang note that their algorithm is very sensitive to the pattern radius *R*, which is dependent on the chosen FOV. The authors compare their algorithm to the grid algorithm and report better performance in terms of magnitude and positional noise, but fail to report the robustness against false stars or star dropout. The algorithm is relatively slow (about 50% slower than the grid algorithm) due to the computationally intensive string matching.

### 3.4. Star Recognition Using the Adaptive Ant Colony Algorithm

**Figure 5:**
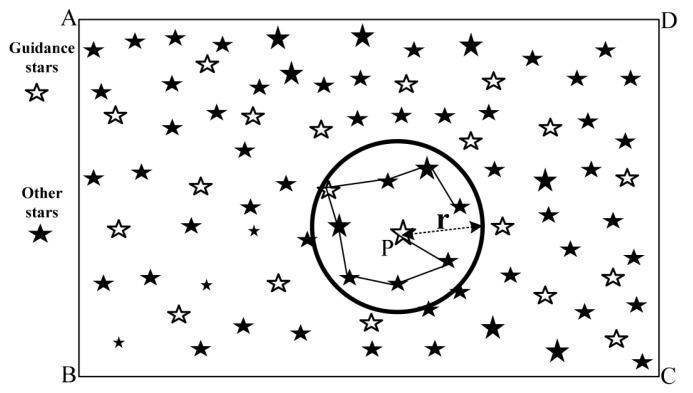
Feature extraction for one star set using AAC algorithm (reprinted from [33]).

Quan and Fang published a novel star identification algorithm in 2010, which uses a pattern based feature extraction by employing an Adaptive Ant Colony (AAC) algorithm [33]. The star identification algorithm is specifically designed to deal with larger FOV (at least 20 * 20 deg) and highly sensitive detectors (no less than 6.9 magnitude). Around a set of stars which have the average grey value of the stars in an image, circles are drawn. The angular distances between all star pairs in this circle are calculated and used to find a unique shortest path that passes through all the stars. The AAC is used to find this path rapidly, as can be seen in Figure 5.

The pattern database is generated in the same way. If the average grey star closest to the centre does not produce a match, the next is tried until a match is found or the set of average grey stars is exhausted, in which case the identification fails. The authors use the binary search method (achieving a search time of O(logn)) for their database search, since the database is sorted on angular distances with ascending order. While the authors compare their algorithm to the Modified Triangle Algorithm [34] and cutting algorithm [35] (both not covered here), they fail to report on the influence of false stars on the star identification rate even though the unique feature pattern is likely to be severely distorted in the case of false stars.

### 3.5. Image Based Identification Algorithms

In 2011, a new development emerged in the field of star identification algorithms. A new pattern based feature extraction approach by Yoon, Lim, and Bang proposed comparing the camera image to a database image by maximising a target cost function [36]. This algorithm is called the Correlation Algorithm. After centroiding, the algorithm reconstructs the original image from the centroid coordinates. However, in order to do so, the algorithm uses the same approach as the grid algorithm (translating and rotating the image around a pivot star by putting the closest star to the pivot star on the *x*-axis). Since this process is likely to fail, the algorithm essentially inherits the same problems as the original grid algorithm. Each star is modelled as a Gaussian distribution with a variance dependent on the system performance and star brightness. The correlation between both images is calculated by multiplying the camera image with a database image, yielding a higher magnitude if the images are more similar. Since this cost function is a cross-correlation of two functions, the authors solve it using a Fourier transform. This process has to be performed for all pivot stars in the database, making the search time linearly dependent on the amount of database entries (O(n)).

**Figure 6:**
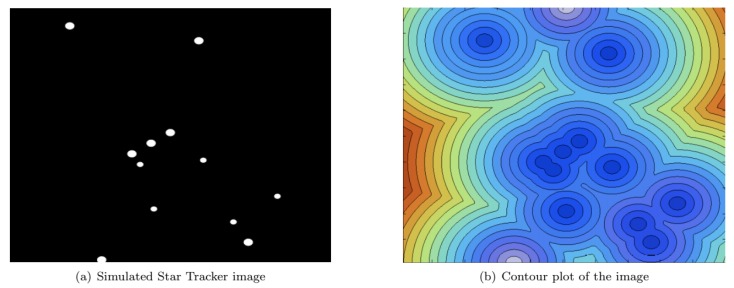
Shortest distance transform feature extraction (reprinted from [3]).

Another image based star identification algorithm was proposed by Delabie, Durt, and Vandersteen in 2013 [3]. The algorithm relies on an image processing technique called the Shortest Distance Transform. A distance transform of a binary image creates a map that colors each pixel according to the distance to the nearest star. This distance is defined as the Euclidean squared distance by the authors. The coordinates of the stars are binned by their pixels, which causes the distance transform map to consist of integers (see Figure 6).

Since the algorithm relies on the comparison of images, the FOV for both the database and the camera needs to be equivalent. The authors generate images of equal sizes evenly distributed over the celestial sphere for the image database. Since the images taken by the star sensor are often rotated and translated with respect to the database images, the algorithm has to solve the same problem as the original grid algorithm: the image has to be rotated and translated in some consistent manner in order to match it to the database images. The authors solve this problem by using the ‘centroid method’. This method translates the database image by matching the centroid of a certain number of brightest stars and rotates it by the smallest angle between the centroid and the stars. This is done for all images in the database, but the authors state that the calculation time for this step is negligible compared to the comparison step. The comparison step (O(n)) consists of creating a distance array consisting of the integer distances of the image stars $N_{tot}$ and the database stars, where $N_{tot}$ is the minimum number of database image and camera image stars. As decision criteria, the sum of the integer distances and the number of database stars within two pixels of the imaged stars are used. These two criteria express how similar the two images are, and the number of close stars divided by $N_{tot}$ is used as an indication of the validity of a solution. Since it is not desirable to compare all the database images to the camera image, a threshold is used on the distance and angle features used in the centroid method to discard around 90% of the images. Furthermore, the centroid and angles are preprocessed and also saved in the database to reduce processing time. While the algorithm is not tested against other algorithms, it is tested for robustness to positional error, false stars and dropped stars. The algorithm is reported to be extremely robust to positional noise and false stars, correctly determining almost 99% of the images with 1000 arc seconds of positional error, and 98% with 650 false stars, respectively. It has to be noted here that the false stars that are added to the image have a magnitude higher than that of the third highest star in the image, which are favourable conditions for testing the algorithm and may not give fair comparisons to other tests. If the three brightest stars are dropped from an image, the algorithm still achieves a 90% matching rate.

The algorithm is implemented for an FOV of 20 degrees squared, which requires 1337 images in the database in order to achieve sufficient robustness. Even though there is a large image overlap in this case, a smaller FOV will lead to an exponentially increased database size. Since the search time is linearly dependent on the amount of images, this limits the algorithm to larger FOV’s.

### 3.6. Pole Star Algorithms Improved

**Figure 7:**
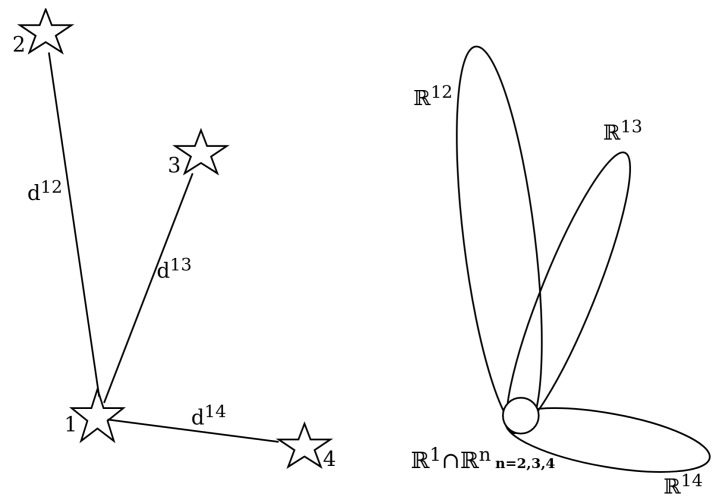
Group match algorithm principle.

Another development has been the further advancement of algorithms based on the *Pole Star* approach. These algorithms have in common that some form of a pole star pattern is used, similar to the original Group Match algorithm put forward by Kosik in 1991 [11]. The algorithms find a pole star and define a number of pairs with the neighbouring stars. Originally, a subgraph isomorphism based feature extraction method, the angular distances are used to find all the matching sets for the star pairs. If there is a star present in all of the found sets, the pole star can be identified (see Figure 7). However, the original approach is not robust to false stars and very storage intensive because it orders the database by angular distance [2]. Attempts have been made to mitigate these shortcomings.

**Figure 8:**
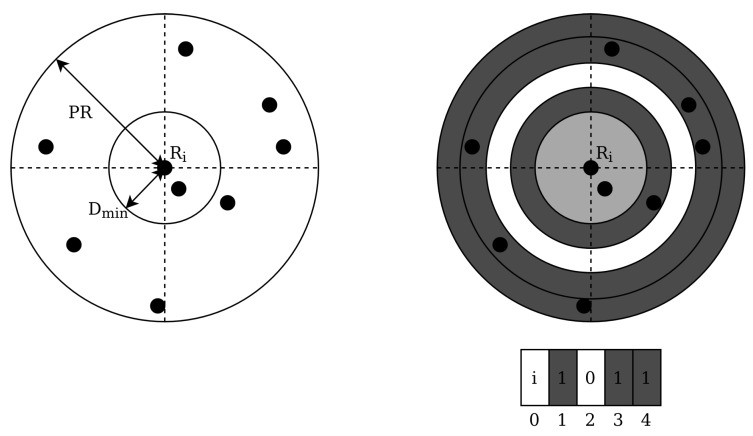
Feature extraction polestar algorithm.

In 2006, Silani and Lovera introduced the Polestar algorithm [37]. The authors define this algorithm as a mix between both feature extraction categories: using a pattern based approach for defining candidates and a subgraph isomorphism based approach for finding a match. The pattern generation scheme is as follows: first, a reference star $R_{i}$ is chosen and the angular distance between this star and all stars outside of a certain radius $D_{min}$ and inside the patter radius PR are calculated. These angular distances are discretised in bins and a binary barcode is generated where a 1 indicates the presence of one or more stars in that bin (see Figure 8).

A set of so-called pattern vectors is combined with all the selected reference stars and a database is generated that is indexed by the binary vector locations. At these index locations, a row constitutes all the stars that match the generated pattern, and a star counter is incremented for each star in the rows that are indexed by the pattern vector with a 1. If the counter is above a defined threshold, the star is considered a candidate. This process voting process is repeated for a number of patterns and has a O(bn) complexity. After the candidate selection step, a subgraph isomorphism feature extraction step is used by generating pairs, triangles and polygons iteratively using the candidate stars. If a match is found between a triangle of a candidate star and the sensor stars, but no match is found for a polygon with a greater number of edges, then an unambiguous identification is made. This step has a complexity of $O(k^{2})$, where k is the number of candidates. Otherwise, no identification is provided. The authors compare their algorithm to the grid algorithm and Liebe’s triangle algorithm [9]. The Polestar algorithm outperforms both in terms of robustness to magnitude and positional noise, but the triangle algorithm outperforms it in terms of speed.

Shortly after, in 2008, Zhang, Wei and Jiang published their algorithm based on radial and cyclic features [38]. This approach is similar to the Polestar algorithm, as the authors also used a binned radial feature pattern and a very similar database structure. However, the subgraph isomorphism step is replaced by a pattern based feature extraction, by generating a bit pattern based on binned cyclic sectors using the angles between the polestar and two other stars. The radial based matching is performed on every star in the image and has O(fn) complexity, where f is the average number of stars in the FOV. The cyclic patterns are generated for every imaged star and compared to the candidate stars achieving O(k). Similar to the Polestar algorithm, the algorithm is only compared to the older grid algorithm, which it outperforms. The cyclic pattern suffers from a similar issue as the original grid algorithm, namely that the selection of the starting side (the side of the smallest central angle) is easily affected by magnitude and positional noise, causing the matching to fail.

Li, Wei and Zhang proposed an iterative algorithm based on a voting mechanism in 2014 [39]. Their subgraph isomorphism based approach consists of three steps: a single match process, an iterative search and verification. In the single match process, the distance between the pole star and one of its neighbours is calculated and used to find corresponding elements within a certain error margin in the database. For each measured star pair, a voting score is increased for both stars if a match is found. If a star counter is above the minimum matching threshold, the database star is considered a match. Generally, multiple candidate stars are found during this process and an iterative search is performed. The candidate stars are used as a reduced stair pair database in the next iteration of the matching process, where the minimum matching threshold is multiplied by the iteration number. If no match is found for a pole star, it is discarded as a false star for the next iteration. The analytical performance of this algorithm can be described as $O(b{\left(\Delta mn\right)}^{2})$, where Δm is the reduced star pair database fraction of the complete database. This analytical performance is equivalent to the analytical performance of the star identification method by Baldini et al. [40] as defined by Spratling and Mortari [1]. If more than two stars have a unique candidate star, a consistency check is performed on the other identified star pairs by checking if the error of the star pairs is within the limits ($O(k^{2})$). After this, a verification step is performed by checking if the number of matched stars is above 4. If this is not the case, the algorithm reports a failed identification. The algorithm is tested against the Group Match algorithm and the Geometric Voting algorithm [41], outperforming both in terms of runtime, robustness against false stars, positional noise and magnitude noise.

**Figure 9:**
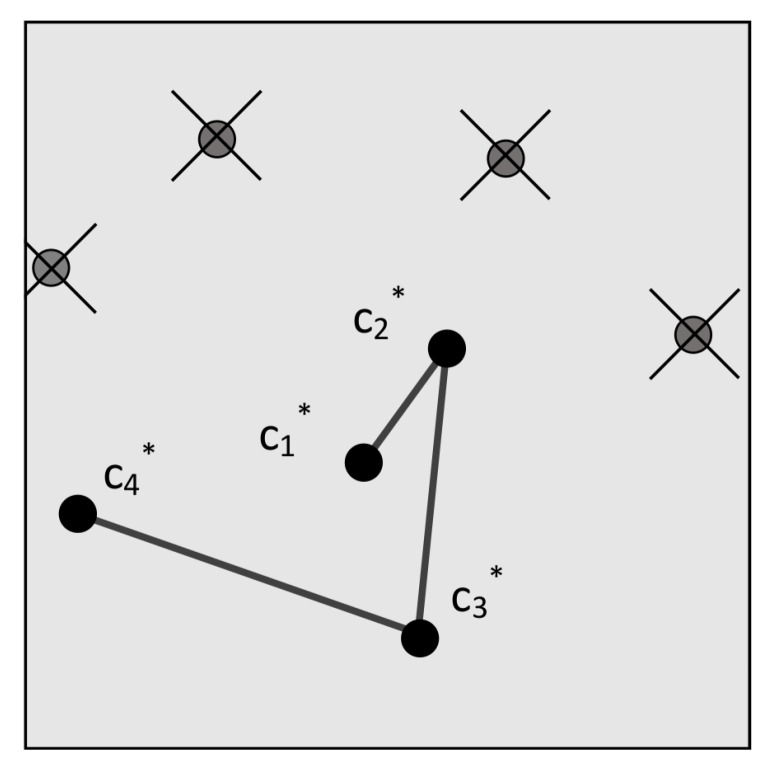
Output of confirmation phase of MPA (reprinted from [42], with permission from Elsevier).

In 2017, Schiattarella, Spiller and Curti introduced the Multi-Poles Algorithm (MPA) [42]. This algorithm also uses a pole star pattern and is specifically designed to be robust against false stars. It uses a subgraph isomorphic feature extraction: the angular distances between the pole star and its neighbours are looked-up in an on-board database using the *k*-vector technique [43] and a list of candidate star sets is returned in O(k). Then, a process very similar to the original group match algorithm is used to ’accept’ the pole star and the neighbour stars, by finding the star that has the largest number of appearances in the sets of candidates. This acceptance phase runs at least twice on different stars in order to reduce the probability of accepting false stars. After this acceptance phase, the verification phase verifies the outcome by cross-checking the sets with each other. In order to mitigate the effects of false stars, a confirmation phase is implemented after the verification phase, which is similar to a method proposed by Xie et al. in 2012 [44]. In this phase, a chain is generated by iteratively checking the angular distances between the pole star and its respective neighbours with the on-board database (see Figure 9). This range search problem is again solved by using the *k*-vector technique and the final output is a chain of confirmed stars that can be used for the attitude determination. The step takes O(bk) since the initial star is already known. While the authors do not compare MPA to other algorithms, they report a 100% identification rate with an input of 185 false stars and 33 cataloged stars.

In 2019, Wei et al. published an algorithm that uses dynamic cyclic patterns that mitigates this single point of failure that is the selection of the starting side [45]. The algorithm uses the discretised centre angles to construct the pattern vector and give this vector a similarity score based on how similar it is to database vectors. While no search method is defined, we assume the search complexity to be O(n). After this, a confirmation phase is implemented using a chaining algorithm, similar to the Multi-Poles algorithm (O(nk)). The authors compare their algorithm to the grid algorithm, the radial and cyclic algorithm, the optimised grid algorithm and the multi-poles algorithm and report better performance in terms of robustness to noise and false stars. However, the performance of their implementation of the Multi-Poles algorithm is significantly worse than what the original authors report due to a different simulation environment.

### 3.7. Deep Learning Approach

**Figure 10:**
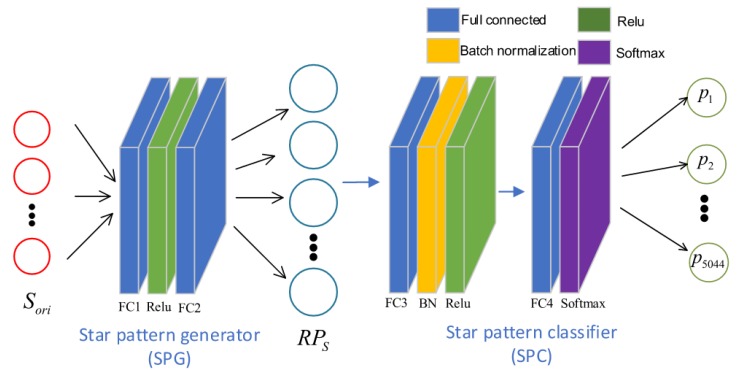
Structure of RPNet (reprinted from [24]).

Deep learning based approaches are not new to the field of star identification algorithms, but they have been limited by the massively parallel processor architecture needed to perform inference. Nevertheless, work continues to be published on this approach and advancements in edge-processing architectures will make deep learning algorithms in space a possibility in the near future. A recent deep learning based approach is RPNet, a star identification network based on representation learning. It was published in 2019 by Xu, Jiang and Liu [24]. RPNet uses a pattern based feature extraction method to construct its input, by selecting a guide star and its neighbour stars and discretizing the distances. Then, an encoder–decoder structure is employed (see Figure 10): a pattern generator is used to create a pattern in a multidimensional space, which is classified using a star pattern classifier. Both the encoder and the decoder need to be trained using artificial star scenes. Once the encoder is trained, its output is used to train the classifier.

The authors compare the algorithm to the grid algorithm and report better robustness to position and magnitude noise and comparable performance in terms of false star robustness. However, the simulation environment has an FOV of 20∗20 degrees, which is relatively large and increases the amount of information the network receives. The algorithm is fast in terms of analytical performance, its inference phase achieves O(1) because the patterns are stored implicitly in the network. The storage size scales with O(n) as the algorithm still requires a lookup database. However, the neural network itself takes up a significant but constant amount of memory.

### 3.8. Summary of Recent Advancements of Lost-in-Space Star Identification Algorithms

In order to comprehensively show a representative view of the recent advancements in lost-in-space star identification algorithms, the covered algorithms are summarised in Table 1. Furthermore, the application environment in terms of a signal-to-noise ratio is listed qualitatively based on the reported robustness to noise, required stars per image and use of verification steps. An algorithm that is able to deal with a lower signal-to-noise ratio can be employed in more challenging application environments. However, usually this does require a more complex solution involving iterative validation. A higher signal-to-noise ratio in a star sensor system may be achieved by a larger FOV, high detector sensitivity, etc. which limits the application environment. The search complexity and validation complexity are also listed since these two complexities are the most time critical and therefore a large driver in the performance of the algorithms.

Clearly a number of algorithms do not have an optimal search strategy which limits the time performance, resulting in linear complexity of the search or worse than linear complexity. The Adaptive Ant Colony and oriented SVD transformation method use a binary search which improves the database search time and greatly improves the performance. Even better is the Multi-Poles Algorithm, which has implemented the *k*-vector technique for the database search. However, this algorithm uses multiple iterations for identification essentially reducing the time performance slightly in favour of reliability. Analytically, the best search performance is achieved by deep learning solutions: because a search is eliminated, the complexity is constant regardless of the size of the problem. However, due to the nature of neural network, an answer is always produced no matter what the input is. This means that proper validation needs to be implemented in order to prevent false positive matching.

Many algorithms do not have a validation step. This is a trade-off in designing a star identification algorithm: while a validation step increases complexity, reliability also increases and the applicable signal-to-noise environment becomes less strict. This can be seen in Table 1: every identification algorithm with a low signal-to-noise application environment has a validation step implemented. The complexity of the validation step is at best O(k), as implemented by Zhang et al. [38]. This step is relatively simple since the search only covers a limited candidate list instead of the whole star database. While the chain validation algorithm requires multiple database searches, it is more robust. The Multi-Poles Algorithm implementation which uses the *k*-vector technique for the searches is the fastest.

### 3.9. Current Challenges and Future Outlook

The majority of research is currently focused on algorithms using a pattern based feature extraction method. Even though these algorithms are fast and resource efficient, the signal-to-noise properties of subgraph isomorphism based approaches are often better. The signal-to-noise tolerance of an algorithm can be improved by including some form of validation in the algorithm, such as the chain algorithm step used in the Multi-Poles algorithm. Since these verification steps are universally applicable, algorithms can easily be adapted to different environments. Incremental improvements of current algorithms to different application environments remain a challenge, especially with regard to improving pattern based algorithms with a form of subgraph isomorphism based verification (producing a hybrid solution such as the Polestar algorithm).

We expect the computing resources in space to increase further. As a consequence, novel approaches that could achieve performance that classical approaches simply cannot achieve will be less restricted by computing resources. Especially deep-learning based approaches, which achieve an analytical performance of O(1), and a linearly scaled database were previously restricted in application by computing constraints. This approach has unrivalled analytical performance and will become a possibility in the near future.

## 4. Comparison Considerations for Star Identification Algorithms

In order to select an appropriate star identification algorithm for a star sensor, a comparison based on a structured approach has to be performed. In their survey paper, Spratling and Mortari evaluate the algorithms by their analytical asymptotic performance in the feature extraction step, the database search and *“their utilization of independent pattern features in the star features based on how many stars are used in a pattern"* [1]. This approach is used because the simulation approach for comparing algorithms does not take into account the sensitivity of the runtime to the size of the data set [27]. While it is true that the difference in relative performance between algorithms is dependent on the analytical performance, it is not a holistic approach in terms of the system performance of a sensor. Since the system has to deal with random noise such as false stars, the analytical performance does not show the full picture to the system engineer as to how well the algorithm performs in real conditions. Therefore, in addition to a comparison based on analytical performance, a simulation approach is often used in literature.

**Figure 11:**
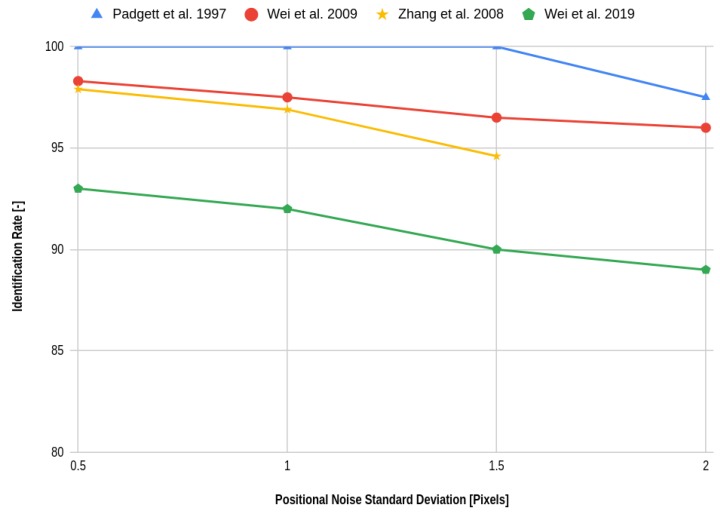
Reported performance of the grid algorithm [7,15,38,45].

While authors frequently report these comparisons, often different performances for the same algorithms under similar conditions are reported across publications. For example, in their paper on the Multi-Poles algorithm Schiattarella, Spiller and Curti specify their application environment as a space environment having a large amount of false objects [42]. The different levels of noise are defined and selected based on a typical mission profile. As stated before, the authors report a 100% success rate under an input of 185 false stars and 33 cataloged stars. However, Wei et al. report that their implementation of the MPA achieves only an identification rate of 91.3% when four false stars are present [45]. This is because Wei et al. use a lower FOV than the original MPA paper and a different detector sensitivity, thereby changing the testing environment. Another example is presented in Figure 11, where the reported performance of the Grid algorithm across literature is shown. While the simulation results are valid, this example shows that quantitative performance claims should not be made on small simulation performance differences. A sensitivity analysis of the underlying robustness is more meaningful.

In 1997, Padgett, Kreutz-Delgado and Udomkesmalee performed a structured comparison of some algorithms representative of the two feature extraction methods [7], evaluating the sensitivity of the algorithms to different testing environments. The authors emphasise that the simulations do not reflect the highest possible performance of the algorithms but rather their purpose is to investigate the underlying robustness of the algorithms. This makes the comparisons more informative and reproducible than presenting differences in performance on different noise levels using the same testing environment. We argue that, when applying a simulation approach, a sensitivity analysis of the performance criteria by varying the testing environment should be performed when using these tests to make general performance claims. To this end, we shall list the considerations for defining the testing environment and the performance criteria here.

### 4.1. Simulation Environment Considerations

Simulation data for the star identification problem are generated using an existing star catalog. For a certain amount of *scenes*, an attitude is generated and all stars within the chosen FOV and magnitude threshold are found in the catalog. An image is generated from this scene using a camera and detector model. Within these models, all types of noise can be added to the image. However, as can be seen in Figure 2, the input to the star identification process are the body-vectors, brightness and accuracy. This means that, in order to mitigate the effects of the star detection and centroiding algorithm on the testing of the star identification algorithm, the input to all algorithms in one test should be equal. Moreover, the test input may consist of a directly generated star scene using a camera and detector model without the need for addressing a detection and centroiding algorithm by simply constructing an array with the centroiding locations and magnitudes.

The main parameters to address when generating the test data are the levels of the different types of noise to add to the scenes. Obviously, for a specific application environment, the relevant noise levels should be investigated and modelled as closely as possible to the realistic values including applicable margins. However, if a qualitative comparison is to be made, a more general approach should be formulated. In order to do so, a testing environment should be specified. Such an environment may include the type of platform for application (small, medium or large satellite), the space environment (levels of radiation, amount of debris, level of FOV obstruction, thermal cycles, etc.), the applicable FOV’s and resolutions of the camera system, the criticality of the star tracker measurements, the intended speed and accuracy levels of the sensor, the maximum body rates (including or excluding jitter), and the level of required camera calibration for the algorithm. All these factors characterise the algorithms application area and dictate the input for the test data generation parameters. Furthermore, most algorithms have certain hyperparameters that should be fixed in order to get consistent results. All chosen parameters should be clearly documented and a sensitivity analysis should be performed on the relevant parameters and their interactions so that the validation space of the comparison is clear.

### 4.2. Simulation Testing Criteria Star Identification Algorithm

For a testing star tracking algorithm, the criteria presented in Table 2 can be identified. A structured comparison does not necessarily cover all the parameters, but should at least clearly specify the testing environment and the reasoning behind the used criteria.

## 5. Conclusions

Since 2009, significant advancements have been made in star identification algorithms; moreover, some truly novel approaches have been proposed. We have identified two categories that cover all feature extraction methods that can be used to classify star identification algorithms. While the majority of current research is focused on algorithms that use a pattern based feature extraction method that is fast and resource efficient, the signal-to-noise properties of current subgraph isomorphism based approaches are better. Hybrid solutions employing a pattern based identification with a subgraph isomorphism based verification phase such as the Polestar algorithm can benefit from the properties of both feature extraction methods.

In terms of signal-to-noise application environment or robustness, the iterative validation techniques used by the Multi-Poles algorithm and the algorithm using dynamic cyclic patterns from 2019 by Wei et al. have proven to provide state-of-the-art performance. The complexity of deep learning approaches such as RPnet is constant (O(1)) because the patterns are implicitly stored in the nodes of the neural network. Analytically, this provides perfect search performance because the database search is eliminated. In terms of space complexity, RPnet also provides analytically perfect performance (O(n)). However, while the memory requirement scales linearly with the number of stars, the neural network itself takes up a significant (but constant) amount of space.

As the industry continues to move to smaller satellites and stricter pointing requirements, star sensors will become smaller and more capable with strong implications on the preferred choice of algorithm. In order to compare these algorithms, a structural approach to testing should be employed that ensures reproducible results. Current literature shows that performance of the same algorithm differs across simulations, which should be taken into account when designing a simulation test. The testing environment should be carefully defined and a thorough sensitivity analysis should be performed by varying the testing environment for quantifying the underlying robustness of the star identification algorithm.

Advancements in technical capabilities such as microprocessors and cameras will make it possible and necessary to apply algorithms in star sensors that were previously too resource intensive to use. Consequently, lost-in-space star identification algorithms with previously unachievable analytical performance such as deep-learning based solutions will be applied in the near future.

## Figures and Tables

**Table 1:** Recent advancements of lost-in-space star identification algorithms.

Author	Year	Feat. Ex.	S/N Environment	Type	Search	Validation
Juang et al.	2003	Pattern	High	Singular Value	O(n)	N/A
Silani and Lovera	2006	Hybrid	Medium	Polestar	O(bn)	$O(bk^{2})$
Na et al.	2008	Pattern	High	Grid	O(bn)	N/A
Zhang et al.	2008	Pattern	High	Polestar	O(fn)	O(k)
Wei et al.	2009	Pattern	Medium	Log Polar Transform	O(n)	N/A
Quan and Fang	2010	Pattern	Very high	Adaptive Ant Colony	O(log(n))	N/A
Yoon et al.	2011	Pattern	High	Image Based	O(n)	N/A
Delabie et al.	2013	Pattern	High	Image Based	O(n)	N/A
Li et al.	2014	Subgraph	Low	Polestar	$O(b{\left(\Delta mn\right)}^{2})$	$O(k^{2})$
Aghaei and Moghaddam	2016	Pattern	Medium	Grid	O(αn)	N/A
Schiattarella et al.	2017	Subgraph	Very low	Polestar	O(k)	O(bk)
Wei et al.	2019	Hybrid	Very low	Polestar	O(n)	O(nk)
Xu et al.	2019	Pattern	High	Deep Learning	O(1)	N/A
Wei et al.	2019	Pattern	High	Singular Value	O(log(n))	$O(k^{2})$

**Table 2:** Scoring criteria star identification algorithm.

Criterium	Definition
Identification Rate	The ratio of scenes in which the **correctly** identified number of stars *i* is larger than a pre-defined number *N* to the scenes where *i*<*N*
Runtime	The average time the identification algorithm takes to identify *N* body vectors in one scene
Storage Size	The amount of storage memory the identification algorithm uses
Memory usage	The amount of Random Access Memory the identification algorithm uses on average
False Positive Rate	The rate of wrongly identified scenes with a positive confidence flag
False Star Robustness	The robustness of the algorithm against the number of false stars *f* in a scene, taking into consideration the ratio of *f* to the number of real stars present in the scene *r*. (Note that real stars not present in the database may be considered as false stars)—usually in terms of identification rate over *f*
Dropped Star Robustness	The robustness of the algorithm against the number of dropped stars *d*, taking into consideration the ratio of *d* to *r*—usually in terms of identification rate over *d*
Positional Noise Robustness	The robustness of the algorithm with respect to the positional noise, usually in terms of identification rate over the average positional noise in a scene
Magnitude Noise Robustness	The robustness of the algorithm with respect to the magnitude noise, usually in terms of identification rate over the average positional noise in a scene
Angular Rate Robustness	The robustness of the algorithm with respect to the simulated angular rate of the sensor—usually in terms of identification rate over angular rate.
Iterations per acquisition	The average number of iterations needed in order to identify N stars.
Complexity	The level of complexity of implementation and maintenance of the algorithm as well as the database structure
